# The human brain—from cells to society

**DOI:** 10.3389/fnhum.2013.00359

**Published:** 2013-08-07

**Authors:** Eva Hoogland, Iain Patten, Stephane Berghmans

**Affiliations:** ^1^Humanities and Social Sciences Unit, European Science FoundationStrasbourg, France; ^2^Scientific Writing ConsultantValencia, Spain; ^3^Medical Sciences Unit, European Science FoundationStrasbourg, France

**Keywords:** human brain, mental health, research policy, translational research

## Abstract

In December 2011, the European Science Foundation (ESF) brought together experts from a wide range of disciplines to discuss the issues that will influence the development of a healthier, more brain-aware European society. This perspective summarizes the main outcomes of that discussion and highlights important considerations to support improved mental health in Europe, including:

1. The development of integrated neuropsychotherapeutic approaches to the treatment of psychiatric disorders.
2. The development of more valid disease models for research into psychiatric disorders.
3. An improved understanding of the relationship between biology and environment, particularly in relation to developmental plasticity and emerging pathology.
4. More comparative studies to explore how scientific concepts relating to the human brain are received and understood in different sociocultural contexts.
5. Research into the legal and ethical implications of recent developments in the brain sciences, including behavioral screening and manipulation, and emerging neurotechnologies.

The broad geographical spread of the consulted experts across the whole of Europe, along with the wide range of disciplines they represent, gives these conclusions a strong scientific and pan-European endorsement. The next step will be to look closely into these five selected topics, in terms of research strategy, science policy, societal implications, and legal and ethical frameworks.

## Introduction

According to recent estimates, ~165 million European citizens will suffer from mental illness in a given year (Wittchen et al., 2011). This equates to around 38% of the European population affected by mental illness alone. Unlike diseases such as cancer or heart disease, the primary burden of brain disorders is linked to disability. Thus, the combination of mental illness and neurological disorders is responsible for around 1 in 3 years of life lost to disability or premature mortality in women and 1 in 4 years in men (Wittchen et al., 2011). Yet despite this enormous societal burden, research investment aimed at the prevention and treatment of brain disorders is much lower than that provided for cancer or other areas of research such as information technology and agriculture (Nature Editorial, 2011; Insel and Sahakian, 2012).

Irrespective of the level of research investment directed toward brain disorders, it has become apparent that, after the first boom of pharmacological treatment possibilities for brain disorders, pharmacological solutions are appearing at a much slower rate than anticipated. The number of new drugs entering the pipeline for the treatment of brain disorders, in particular mental illness, has declined dramatically (Miller, 2010). As a result, there is now a dramatic discrepancy between what is needed and what is being done to meet that need. Yet hope is to be found in fields such as psychotherapy. Psychotherapy has made tangible advances over the last 10–15 years, and in many cases such treatment is at least as effective as drug therapy, while in some disorders the combination of pharmacological treatment and psychotherapy has demonstrated the best results (Cuijpers et al., 2011).

Understanding brain function is not only of use to medicine—it is important for all aspects of individual health and wellbeing. Many psychiatric disorders are known to begin during childhood and adolescence, at a time when brain plasticity is also critically important to learning and socialization, for instance. Insights into both healthy development and pathology could therefore have implications that extend well beyond the treatment and prevention of disease. In fact, insights into brain function are now beginning to raise important questions about how we determine legal responsibility or how we understand the processes underlying economic decision-making.

Despite the wide-ranging importance of the brain sciences, there is a widespread lack of awareness of the issues at stake. Societal understanding of neuroscience research is both limited and plagued by misconceptions (Racine et al., 2005). But public understanding is not the only problem area. Institutions from schools to courts are increasingly in need of reliable information on brain function and its implications in their specific areas of interest. Likewise, researchers in the various different fields that make up the brain sciences would all benefit from a greater awareness of their respective contributions and viewpoints.

This perspective is based on discussions held during a 1-day workshop in Berlin in December 2011 as part of the European Science Foundation (ESF) strategic initiative *The Human Brain: From Cells to Society*. The meeting brought together experts in fields ranging from philosophy and anthropology through clinical neuroscience to cellular and molecular neurobiology. The document is intended to provide a first step in a long-term discussion of future research and practice in light of the changes occurring in our understanding of the human brain. It should be emphasized that it has not been the intention of the current paper to provide specific recommendations for action. Rather, it is a first step designed to narrow the overarching field of “The Human Brain—from Cells to Society” to a limited number of specific topics that are of current relevance due to their societal implications or because they hold particular promise for important scientific breakthroughs. The list of recommended topics that you will find at the end of the report is a reflection of the agreement reached by the participants. The broad geographical spread of the consulted experts across the whole of Europe, along with the wide range of disciplines they represent, gives these conclusions a strong scientific and pan-European endorsement. The next step will be to look closely into these five selected topics, in terms of research strategy, science policy, societal implications, and legal and ethical frameworks. Only after these carefully managed further steps, will we be able to expect thorough recommendations. The ESF initiative “The Human Brain—from Cells to Society” intends to facilitate this process and, where possible, to support scientists and member organizations in that endeavor. We encourage all stakeholders concerned to join us and to take up this challenge.

## Levels of organization—levels of understanding

The human brain can be understood on a number of levels, from the genes that control its development and physiology through to the behavior it generates and even beyond to social and cultural phenomena. These different levels are often understood in terms of a functional hierarchy. Thus, gene expression determines the molecular composition of the brain, which in turn defines the basic building blocks for the cells that will regulate its physiology. At the next level, neuronal connectivity, defined by synaptic interactions, underlies the establishment of microcircuits and, ultimately, the gross connectivity of brain regions. How these levels of organization translate into complex behavior is only just beginning to be understood, yet it seems clear that this brain organization at least provides the foundations for behavioral expression.

Such a hierarchy is also reflective of the approaches used to investigate the brain. The genetic research community, for instance, has focused on identifying genes that control the differentiation and connectivity of neurons in the developing brain, as well as those gene variants that are associated with specific behaviors or neuropsychiatric disorders. Similarly, neuroanatomists and physiologists have explored the role of different brain areas in controlling specific functions and psychologists have sought insight into the behavioral interactions between individuals within a social or cultural context. Focusing research on one level, however, can restrict our capacity to achieve a truly mechanistic understanding of the brain.

Many of the basic building blocks of the brain, in terms of genetic and molecular components, are now understood. The human genome is sequenced and many of the products of gene expression are characterized. Yet the way in which these components influence the behavioral output of the human brain are largely limited to associations between gene variants or neurochemical profiles (e.g., levels of monoamine neurotransmitters) and behavioral disorders (Burmeister et al., 2008; Shyn and Hamilton, 2010). The challenge for the future will be to gain insight into how those genes affect the cellular composition and synaptic organization of the brain, and how this determines the organization of microcircuits and higher-level regional organization and connectivity. The same principles apply to research focused on other levels such as synaptic physiology or microcircuits.

We must be wary of taking a unidirectional, biological reductionist view in our attempts to understand brain function, however. In some areas of the brain sciences, the principle that biology influences behavior is well accepted without a similar recognition of the effects of psychosocial interactions on biology. Yet psychosocial interactions such as maternal support in childhood are already known to influence brain structure (Luby et al., 2012). Just as each step must be understood from genes and molecules to behavior and social interaction, therefore, so must the effects of psychosocial interactions be traced back (Hein et al., 2010).

## Expanding views of development and plasticity

Since antiquity, philosophers and scientists have debated the role of nature and nurture in the development of human behavioral and cognitive features. The challenge for the brain sciences is therefore now to embrace an expanded view of development and plasticity that focuses on gene–environment interactions (Bendesky and Bargmann, 2011). Examples of the shifting view of acquired and innate characteristics in the developing human brain can be found in research into language development in human infants. Comparison of cry patterns in new-born infants exposed to German or French in the womb, for instance, indicates that the prosodic features of the language are present in infant cries (Mampe et al., 2009). Thus, an acquired feature of language is already apparent at birth. Such features, however, are thought to be dependent upon a biological predisposition for melody perception and production (Mampe et al., 2009). Thus, even before birth, a clear distinction between nature and nurture is difficult to draw.

Whether a characteristic is acquired prenatally or postnatally, it is clear that certain features of language and cognition usually develop at a certain stage. This has led to the view that there are windows of opportunity during which a characteristic becomes fixed (Kuhl, 2010). This view is influenced by the observation of critical periods during which features such as visual perception become established (Wiesel and Hubel, 1963). But research has now begun to question this linear view. In language development, for instance, Japanese adults who have had limited exposure to English are generally understood to have lost the capacity to contrast between /r/ and /l/ phonemes. Thus, once a critical period or window of opportunity has passed, those individuals will no longer be able to learn and reproduce this distinction. Recent studies have shown, however, that the distinction can still be learnt under the right conditions (Zhang et al., 2009). Thus, previous assumptions about the limits of developmental plasticity may not always hold.

The realization that critical periods for the development and acquisition of neural functions may not be as fixed as once thought suggests that it is now time to take a wider view of development and plasticity. It is time for research to move beyond looking at infants alone and seek to understand more clearly what happens between infancy and adulthood. This is of relevance not only to cognition and language but also to emotional and psychosocial development. Many psychiatric disorders, for instance, are understood to have their origins in puberty, yet very little is understood about what actually happens to the brain during this period (Paus et al., 2008). If we can improve our understanding of developmental processes and potential pathology across a much wider age range, we will increase the opportunity for early intervention and preventive strategies. Ultimately, this will require long-term longitudinal studies (Schumann et al., 2010). Importantly, if we are to begin to understand the relationship between the environment and biological processes, such studies will need to encompass all levels of understanding, from genes to social interaction.

## Translating knowledge into practice—treatment and prevention of brain disorders

The treatment of mental illness has for decades been highly polarized. These two sides could be described as “bottom-up” and “top-down” but we prefer the more classical terminology of “biological” and “psychological.” Even though this terminology may be considered slightly naive, it emphasizes that these different approaches stem from different disciplines and have a whole different academic tradition behind them. This disciplinary issue is key to the current paper. One side in the debate, which we will refer to as “biological psychiatry,” has championed the view that mental illness has an organic basis and that treatment must therefore be focused on physical (generally pharmacological) correction of a biological defect. The other side, primarily psychosocial psychiatry and psychology, has focused on the psychological causes of disturbance and sought psychological solutions to correct it. The forceful opinions expressed by both sides reflect a strong ideological division that largely continues the mind–body dichotomy that has fueled philosophical debate for centuries.

The separation of biology and psychology in our understanding of the causes and treatment of mental illness also highlights major gaps in our understanding of brain function. As we move away from a biological reductionist view of the human brain, we can begin to explore how psychosocial interactions influence the structure and function of the brain in the same way as its genetic, molecular, and cellular organization can regulate cognition and psychology. As a result, we can begin to understand psychosocial interventions not only in terms of their psychological effects but also their influence on the organic structure and physiology of the brain. This could prove to be a particularly fruitful avenue of exploration.

Following the major advances that were made in the psychopharmacological treatment of mental illness, psychotherapeutic approaches—by which we understand a wide range of therapeutic interactions or treatments, including psychiatry, clinical psychology, counseling psychology, occupational therapy and psychoanalysis—have now begun to show effect sizes that are equal or superior to pharmacotherapy in many disorders (Cuijpers et al., 2011). Moreover the combination of the two approaches has proven to be the most effective in major chronic psychiatric disorders such as bipolar affective disorder or schizophrenia. Randomized controlled trials in bipolar disorder have shown that maintenance pharmacological treatment and psychotherapeutic interventions in combination had the best effect on long-term outcomes, such as relapse or rehospitalization (Vieta et al., 2009). Another example is in the treatment of borderline personality disorder. Reviews of current evidence suggest that pharmacotherapy may be useful for the treatment of individual symptoms, but it is not an effective approach to reducing the overall severity of borderline personality disorder (Lieb et al., 2010). In contrast, preliminary findings in small studies have supported the potential efficacy of psychotherapeutic approaches (Binks et al., 2006). More recent trials have continued to show evidence supporting the efficacy of psychotherapeutic interventions (Bateman and Fonagy, 2008, 2009; Farrell et al., 2009; Paris, 2009). It remains an open question, however, exactly what effect these approaches have on the brain. Interestingly, the specificity of a therapeutic intervention can be considered an open question for both psychotherapy and pharmacotherapy. Any effect on one part of the brain can be assumed to affect the brain as a whole. Greater insight is therefore required into both the specific and wider consequences of any therapeutic intervention.

As we develop insights into the effects of psychotherapy and psychosocial interventions on the brain, it should be possible to tailor the treatment to individual needs and develop a truly neuropsychotherapeutic approach. In the short term, however, there are already steps that can be taken toward an integration of biology and psychology. For instance, pharmacological interventions are now becoming available that could be used to facilitate the use of psychotherapy in a variety of psychiatric disorders. It has been suggested that the use of certain neuropeptide drugs, such as oxytocin and vasopressin, could facilitate interaction-based psychotherapy for disorders involving early attachment disruption or abnormal social interaction such as social anxiety disorder and borderline personality disorder (Meyer-Lindenberg et al., 2011). Such approaches are particularly exciting given the enormous difficulty associated with the treatment of social disorders. They also highlight an overall principle of combining biological and psychological interventions to enhance the potential efficacy of treatment. In the next 10 years, it can reasonably be expected that substantial advances will be achieved in this way.

The longer-term goal of research into the treatment of brain disorders is of course to move away from symptomatic treatments and toward therapies that target the underlying etiology. The hurdles that must be overcome to move beyond symptomatic treatment in psychiatric disorders are particularly challenging, however, since we must first develop a much more detailed understanding of the etiology of the disorders. In conditions such as schizophrenia, for instance, only very fragmented information is available on the underlying pathology and even less on the mechanisms leading to the development of symptoms (Stöber et al., 2009). Recently, findings from various fields have begun to be synthesized to show that dysfunction of inhibitory interneurons might be a final common pathway that leads to divergent symptoms in schizophrenia and other disorders (Marin, 2012). Continuing such research efforts aimed at understanding the underlying pathophysiology of brain disorders will be of far more than merely academic interest—it is absolutely crucial to their future treatment and prevention.

Experimental testing of therapeutic interventions is heavily dependent upon the use of animal models under clearly defined conditions. Most psychiatric disorders are diagnosed based on a constellation of symptoms (American Psychiatric Association, 2000), and this presents major problems for the establishment of reliable animal models. It is unlikely, for instance, that a single animal model will unite all of the symptoms required for the diagnosis of complex psychiatric disorders such as schizophrenia, depression, or personality disorder. The focus must therefore be on developing models that reflect the pathophysiology of brain diseases. One important step toward this goal will be the identification of definitive biomarkers for psychiatric disorders, and this will also offer clear clinical benefits for improved diagnosis. Another avenue of interest for psychiatric research is the development of *in vitro* disease models based on induced pluripotent stem cells, which will also serve to identify biomarkers and molecular disease pathways (Brennand et al., 2012).

## Toward a brain-aware society—dealing with the implications of advances in the brain sciences

Many advances in biomedical research have had social and societal implications. Perhaps the best example is that of genetics, where much debate has arisen around privacy and (mis)use of personal information (Clayton, 2003). The various disciplines that together form the brain sciences, however, merit specific consideration. Since research in this area touches on areas such as identity, free will, and responsibility, it has the potential to influence the very way in which we see ourselves as human beings. As a result, the impact of the brain sciences extends far beyond health and education and includes areas such as legal responsibility, treatment vs. enhancement, military applications, and the ethical limits of behavioral assessment.

The identification of biomarkers to facilitate the diagnosis of psychiatric disorders has important implications (Singh and Rose, 2009). Biomarkers are not only indicators of pathology; they also have the potential to predict susceptibility to illness. Thus, if we were able to recognize early pathophysiological signs of a disease such as schizophrenia in children, we might ultimately be able to avert its course. But there are also significant dangers of the indiscriminate or ill-informed use of biomarkers for behavioral traits. The same biomarkers that are used for diagnosis or risk stratification of psychiatric disorders could in principle be used to identify individuals who are likely to display the behaviors or personality traits that define them.

Screening for individual biomarkers of behavioral traits could focus attention on the individual and away from social and environmental factors (Singh and Rose, 2009). Many childhood behavioral problems, whether or not classified as specific disorders, are thought to have links with youth and adult criminality or antisocial behavior, for instance. This is the case for psychiatric diagnoses such as attention deficit hyperactivity disorder, where concerns have already been raised about the risk-benefit ratio of the use of medication and the process of medicalization in very young children (Singh and Rose, 2009). Categorization of children as potential future delinquents carries with it the potential to alter their perception of themselves and the way that they are treated by others at a very early stage in their life trajectories. We must therefore ask ourselves whether we have sufficient insight into the potential neuropsychological effects of this sort of early risk prediction. For instance, how will a child who is identified as at increased risk of future antisocial behavior or criminality be treated by those responsible for his or her welfare? Likewise, how will a child's self-image be affected by this knowledge and by the resulting changes in behavior that might occur in caregivers and other significant adults? These and other related issues must be considered carefully to avoid potentially helpful information having unexpected or even obviously damaging consequences. Furthermore, similar questions apply to screening for learning deficits and early cognitive traits applicable to child education and social development.

Concerns about discrimination and stigmatization of individuals identified as being at risk for future psychiatric illness or as already having neuropsychological abnormalities highlights a current concern over the potential misuse of the brain sciences. On the one hand, evidence suggests that there is a great deal of plasticity in brain function and that even apparently “fixed” traits can be changeable under the right conditions. Yet on the other hand, public perception and even views held among professionals can reflect a powerfully deterministic view of behavior. According to such a deterministic view, someone who carries biomarkers for future behavioral traits or mental illness is at risk of discrimination rather than being provided with an opportunity for support and intervention that allows positive change. Of course, intervention itself can be either supportive or coercive. The potential for social control based on behavioral norms, even when a non-deterministic view of behavior is adopted, is clearly quite substantial. As has been argued elsewhere, the only way in which to understand the social implications of a biomarker is to undertake detailed qualitative research in a wide section of the population (Singh and Rose, 2009). The findings of such research will allow policies to be established that maximize the benefit and minimize the potential harm associated with the introduction of biomarkers for psychiatric disorders.

Questions of determinism and plasticity also influence our view of legal and social responsibility (Freeman, 2011; Buchen, 2012). According to a deterministic view of behavior, individuals could be deemed as not responsible for their actions if it is shown that their brain structure or physiology, for example, is associated with a particular criminal behavior. Equally, those who carry biomarkers of behavioral traits such as propensity to violence could be at risk of being detained or controlled pre-emptively in a society that is increasingly unwilling to accept perceived risk (Eastman and Campbell, 2006; Buchen, 2012). If probabilistic indicators of predisposition are mistakenly interpreted as biological determinants, we risk seeing them as functioning entirely in the absence of other environmental and psychological factors. Under such conditions, the risk of neuroscience being used as a tool to support oppressive social policies is very real.

Given such risks, can we reasonably expect juries and the legal profession to be sufficiently versed in neuroscience, or indeed science in general, to understand the nature and reliability of the evidence presented to them? Scientific evidence is usually employed in law to determine whether an individual did or did not commit a crime. In the case of behavioral neuroscience, however, the purpose of the evidence is to decide whether the defendant had wrongful intent (Eastman and Campbell, 2006). Under these conditions, neuroscientists should perhaps be even more wary of how their expert status could be misused or exploited. If inappropriate responsibility is given to neuroscientists as arbiters of individual intent, there is a risk of returning to a situation somewhat akin to opinions on the goodness of someone's *élan vital*. The scientific community therefore has a responsibility to ensure public understanding of the potential roles and limitations of neuroscience research in legal and social contexts.

If we are to make practical use of scientific concepts, we must understand the sociocultural context in which they are received and understood. On one level, the influence of neuroscience will be culturally dependent, as has been observed in relation to other areas of biomedical science such as immunology (Martin, 1994). The concept of cultural dependence, however, can also apply to knowledge communities. How, for instance, do core concepts differ between neurobiology and psychology, or between social psychology and anthropology? Ultimately, the importance of understanding the human brain requires that disciplinary communities be brought together to reach a common goal. Yet this requires the different groups to be able to communicate effectively with each other. There is currently very little information available on how key concepts such as empathy are understood in different knowledge communities (e.g., brain imaging and social psychology). Indeed, anecdotal evidence suggests that some fields are hostile toward the approaches and thinking of others. The effectiveness of future research will therefore be dependent on identifying ways to ensure that interdisciplinary efforts lead to cross-fertilization rather than cross-sterilization.

Any proposed brain-aware society must reflect the different ways in which we understand ourselves as human beings. This can be described in positivist, interpretational, and phenomenological terms. Positivist descriptions focus on the underlying cause of a psychological phenomenon, interpretational descriptions on our beliefs about the cause of the phenomenon, and phenomenological descriptions on our reasons for those beliefs. Neuroscience, however, currently focuses almost exclusively upon positivist descriptions. For instance, by attempting to describe human behavior in terms of underlying biological or neurochemical changes, it seeks to provide an underlying physical cause for psychological phenomena. If society as a whole is to embrace neuroscience and become more brain aware in its approaches to education, the legal system, and social responsibility, discussion must also leave room for interpretational and phenomenological understanding.

Finally, a brain-aware society must also be equipped to deal appropriately with developing technologies. The widespread use of techniques such as functional magnetic resonance imaging and positron emission tomography has yielded important insights into brain function. Likewise, technologies such as cochlear implants have been of enormous benefit to large numbers of people. More recently, opportunities have developed for neurorobotics and brain stimulation to play important roles in medical or other applications. However, with these developments, we must now begin to address the social implications of tools that could allow information not only to be read from the brain but also perhaps written back into it (Wolpe et al., 2010; Heinrichs, 2012). The potential for such technologies to invade the integrity and freedom of the individual is quite real. Society may need to determine, for instance, what belongs to the individual and what can be decoded in the public interest. Likewise, the potential use of brain stimulation to introduce information into the human brain or enhance its function will require careful ethical monitoring. These and other questions, such as military applications of neurotechnologies (Brain Waves Module, 2012), are in need of urgent debate at all levels of an emerging brain-aware society.

## Conclusions and future directions

In summary, discussions among participants highlighted five key opportunities for important advances to be made in our understanding of the human brain, from cells to society (see text box). The first opportunity expresses the overarching biomedical goal for which the two subsequent opportunities are required. Opportunities 4 and 5 fall under another banner addressing very different but equally important and far-reaching issues related to societal challenges and impact. Each of these five areas is expected to capitalize on existing research strengths in Europe while also embracing the broad relevance of the brain sciences to society.

This should be taken as the first step in a long-term process. The next step will be to look closely into the five selected topics, in terms of research strategy, science policy, societal implications, and legal and ethical frameworks. This should lead to specific recommendations that allow for an effective implementation. The ESF initiative “The Human Brain—from Cells to Society” was established to facilitate this process and, where possible, to support scientists and member organizations in that endeavor. We encourage all stakeholders concerned to join us and to take up this challenge.

### Conflict of interest statement

The authors declare that the research was conducted in the absence of any commercial or financial relationships that could be construed as a potential conflict of interest.

## Steering committee

Professor Ina Bornkessel-Schlesewsky

Department of Neurolinguistics, Philipps-Universität Marburg

Professor Daniel David

Babeş-Bolyai University, Cluj-Napoca

(Standing Committee for the Social Sciences)

Professor Henry Makram

École Polytechnique Fédérale de Lausanne, Switzerland

Professor Csaba Pléh

Budapest University of Technology and Economics

(Standing Committee for the Humanities)

Professor János Réthelyi

Semmelweis University, Budapest

(European Medical Research Councils)

Ms Malgorzata Tkatchenko

Commissariat à l'Energie Atomique, Gif-sur-Yvette

(Standing Committee for the Physical and Engineering Sciences)

Professor Đur**đ**ica Ugarkovi**ć**

Ruder Boskovic Institute, Zagreb

(Standing Committee for the Life, Earth and Environmental Sciences)

## Meeting participants

Professor Stefan Beck

Institut für Europäische Ethnologie, Berlin

Professor Jean-Pierre Changeux

Laboratoire de Neurobiologie Moléculaire, Institut Pasteur/Collège de France

Professor Carsten de Dreu

Department of Psychology, University of Amsterdam

(President European Association for Social Psychology)

Professor Luciano Fadiga

Department of Human Physiology, University of Ferrara

Professor Angela Friederici

Max-Planck-Institute for Human Cognitive and Brain Sciences, Leipzig

Professor Sten Grillner

Nobel Institute for Neurophysiology, Karolinska Institute, Stockholm

(President Federation European Neuroscience Societies)

Professor John-Dylan Haynes

Bernstein Center in Computational Neuroscience, Berlin

Ms Ira van Keulen

Department Technology Assessment, Rathenau Institute, The Hague

Professor Ilona Kovacs

Department of Cognitive Science, University of Technology and Economics, Budapest

Professor Uskali Mäki

Department of Philosophy, University of Helsinki

Professor Andreas Meyer-Lindenberg

Central Institute of Mental Health, Mannheim

Professor Edvard Moser

Kavli Institute for Systems Neuroscience, Norwegian University of Science and Technology

Professor Andreas Roepstorff

Center of Functionally Integrative Neuroscience/Department of Anthropology, University of Aarhus

Professor Frank Rösler

Department of Experimental Psychology, University Potsdam

Professor Josef Syka

Department of Auditory Neuroscience, Charles University Prague (European Medical Research Councils)

Dr Virginie van Wassenhove

Cognitive Neuroimaging Unit, CEA/DSV/I2BM/NeuroSpin, Gif-sur-Yvette.
